# Detection and delineation of oral cancer with a PARP1 targeted optical imaging agent

**DOI:** 10.1038/srep21371

**Published:** 2016-02-22

**Authors:** Susanne Kossatz, Christian Brand, Stanley Gutiontov, Jonathan T. C. Liu, Nancy Y. Lee, Mithat Gönen, Wolfgang A. Weber, Thomas Reiner

**Affiliations:** 1 Department of Radiology, Memorial Sloan Kettering Cancer Center, New York, NY 10065, USA; 2 Department of Radiation Oncology, Memorial Sloan Kettering Cancer Center, New York, NY 10065, USA; 3 Department of Mechanical Engineering, University of Washington, Seattle, WA 98195, USA; 4 Department of Epidemiology and Biostatistics, Memorial Sloan Kettering Cancer Center, New York, NY 10065, USA; 5 Department of Radiology, Weill Cornell Medical College, New York, NY 10065, USA; 6 Molecular Pharmacology Program, Memorial Sloan Kettering Cancer Center, New York, NY 10065, USA

## Abstract

Earlier and more accurate detection of oral squamous cell carcinoma (OSCC) is essential to improve the prognosis of patients and to reduce the morbidity of surgical therapy. Here, we demonstrate that the nuclear enzyme Poly(ADP-ribose)Polymerase 1 (PARP1) is a promising target for optical imaging of OSCC with the fluorescent dye PARPi-FL. In patient-derived OSCC specimens, PARP1 expression was increased 7.8 ± 2.6-fold when compared to normal tissue. Intravenous injection of PARPi-FL allowed for high contrast *in vivo* imaging of human OSCC models in mice with a surgical fluorescence stereoscope and high-resolution imaging systems. The emitted signal was specific for PARP1 expression and, most importantly, PARPi-FL can be used as a topical imaging agent, spatially resolving the orthotopic tongue tumors *in vivo*. Collectively, our results suggest that PARP1 imaging with PARPi-FL can enhance the detection of oral cancer, serve as a screening tool and help to guide surgical resections.

Oral cancer, the most common form of head and neck cancer, is defined as a malignant neoplasm of the lip, oral cavity, and pharynx. In 2014, more than 40,000 patients in the United States and almost 500,000 patients worldwide were diagnosed with the disease^1^. Oral cancer can often be effectively managed when diagnosed at a localized stage, but almost two thirds of all patients present with advanced disease, resulting in poor survival (62% and 38% five-year survival for regional and distant disease, respectively) and speech, swallowing, and cosmetic problems. Although there is a strong clinical need to improve the early detection of oral cancer, current diagnoses are still almost exclusively based on visual inspection of the oral cavity, followed by biopsy of suspicious areas^2^.

Targeted optical imaging probes have the potential to add another layer of information to standard of care patient examinations. The molecular information provided by these agents complements visual inspections, identifies suspicious areas in the oral cavity and facilitates diagnosis, screening and intraoperative protocols^3^,^4^. Successful targeted optical imaging requires the identification of a robust biomarker for oral cancer, paired with its successful validation as a target for a molecular imaging probe^2^,^5^,^6^.

More than 90% of all cancers of the oral cavity are oral squamous cell carcinoma (OSCC)^7^, a type of cancer that originates in the oral mucosal lining of the oral cavity. This anatomic feature suggests the use of a fluorescent imaging agent for this type of malignancy^8^,^9^ because the agent can detect superficial tumors with high sensitivity and provide high-resolution, real-time images^10^,^11^,^12^,^13^. A biological target that could enable OSCC imaging is the nuclear enzyme Poly(ADP-ribose)Polymerase 1 (PARP1). PARP1 is a valuable therapeutic target in pharmaceutical research due to its critical role in DNA repair^14^,^15^,^16^. However, because it is overexpressed in a multitude of different cancers, PARP1 could also be a high-value target for the detection, staging, and biological characterization of cancer^17^,^18^,^19^,^20^,^21^,^22^,^23^,^24^.

We have shown in models of cancer that PARPi-FL, a fluorescent PARP1-targeted small molecule, specifically binds to PARP1 with a similar affinity to Olaparib (Lynparza, Astra-Zeneca), an FDA-approved PARP1 inhibitor. These studies were performed in cell culture and with *ex vivo* imaging of excised tumors. Here, we test the hypothesis that fluorescent imaging with PARPi-FL can detect and delineate OSCC *in vivo*. We first determined the expression of PARP1 in human OSCC using biospecimens from OSCC patients, and identified mouse models of OSCC that reflect the human disease, including the expression levels of PARP1. We then tested PARPi-FL in these mouse models and confirmed that clinically relevant, non-invasive imaging systems are capable of visualizing OSCC with high contrast after both intravenous and topical administration of PARPi-FL, suggesting that PARPi-FL could be used to answer diagnostically relevant questions in the clinic.

## Results

### PARP1 expression in human oral cancer biospecimens

**Figure 1:**
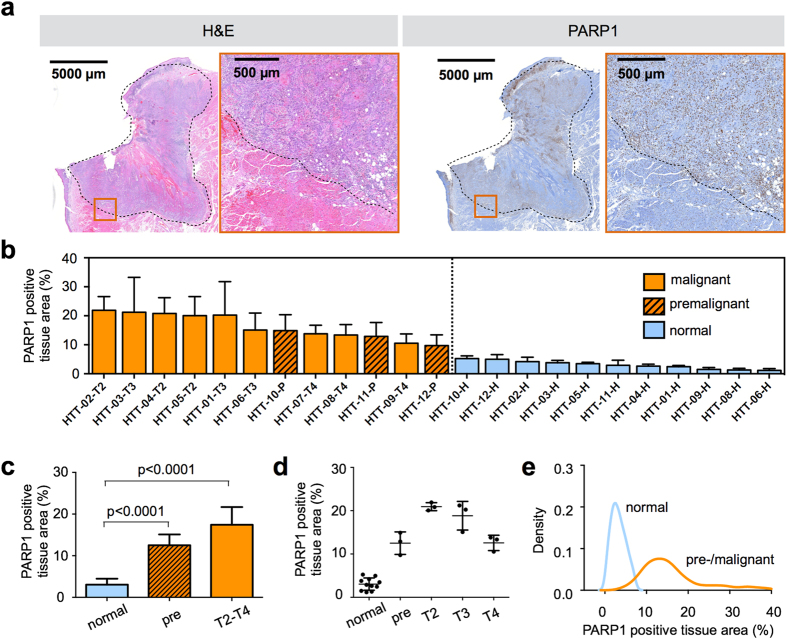
PARP1 expression in human tongue tumors. (**a**) Surgically removed human squamous cell carcinoma specimens of the tongue were stained for PARP1 using an anti-PARP antibody and immunohistochemical detection. Adjacent sections were stained with H&E for pathological evaluation and staging of the malignancy of the tissue. The dotted line marks the tumor margin in the displayed T2 staged tumor. The red squares indicate enlarged regions in adjacent images. The image shown is a representative example selected from n = 12 samples. (**b**) PARP1 quantification in human tongue tumor tissue in a waterfall plot of the PARP1-positive tissue area. The PARP1-positive tissue is represented by brown (PARP1) and the total tissue areas by blue staining. In each sample, 5–10 fields of view in the tumor area or adjacent healthy tissue were analyzed. Displayed are means ± SD. HTT = human tongue tumor; color code: orange = malignant tissue (squamous cell carcinoma T2-T4), dashed orange = premalignant cases (moderate to severe dysplasia, carcinoma *in situ*), blue = corresponding normal adjacent tongue tissue. (**c**) PARP1-positive tissue area, grouped for normal tissue, premalignant, and malignant (T2-T4 tumor stages) cases. Statistical significance was determined using an analysis of variance where multiple comparisons were controlled using the Holm-Šidák at the familywise error rate of 5%. (**d**) Individual values for PARP1-positive tissue area, grouped for the pathologically assessed tumor stage (premalignant, T2, T3, T4) and compared to normal adjacent tissue. (**e**) Density plot of all PARP1-positive tissue area values from each field of view, pooled in two groups (normal and premalignant/malignant). See Supplementary Fig. S2 for additional statistical parameters.

To determine the relevance of PARP1 as a biomarker for OSCC, we first explored PARP1 expression patterns in human oral cancer tissues, along with PARP1 expression in adjacent healthy tissues in 12 human tongue tumor specimens obtained from the Department of Pathology at Memorial Sloan Kettering Cancer Center (MSK), which were histopathologically staged using H&E stained biopsy tissue following the standard tumor, node, metastasis (TNM) classification. The tissues included three specimens per tumor stage: premalignant, T2, T3, and T4 (Table S1). The premalignant tissues were classified as moderate/severe dysplasia and squamous cell carcinoma *in situ*. All tissue specimens except for one were obtained from the edges of the tumors, and featured both tumor tissue as well as healthy surrounding tissue (Fig. 1a). In all cases, intense PARP1 expression as determined by immunohistochemistry (IHC) clearly distinguished tumor from adjacent normal tissue (Fig. 1 and Supplementary Fig. S1). In normal tissue, the PARP1-positive area on IHC ranged from 1.2% to 5.3%, whereas for all malignant and premalignant specimens, it ranged between 9.7% and 21.2%, with almost no overlap between the two groups (Fig. 1b and Supplementary Fig. S2a). The mean PARP1-positive area was 3.1 ± 1.4% in normal tissue, 12.6 ± 2.5% (P < 0.0001 vs. normal) in premalignant tissue, and 17.4 ± 4.2% (P < 0.0001 vs. normal, Fig. 1c) in malignant tissue.

We also observed differences in PARP1 expression when comparing different tumor stages, albeit these are based on small sample sizes (n = 3 per tumor stage) (Fig. 1d). T2-staged tumors featured the highest PARP1 expression (21.9 ± 1.0%), followed by T3-staged specimens (18.9 ± 3.3%), T4-staged tumors (12.6 ± 1.8%), and premalignant tissues (12.5 ± 2.6%). Pooling all PARP1 expression values of malignant and normal specimens (5 to 10 values per specimen) in a density plot shows that the overlap between malignant and normal populations was very small (Fig. 1e). The variation of the PARP1-positive area values in normal tissue resulted in a narrow symmetric density plot between 0 and 10% PARP1-positive area, whereas malignant tissue showed a broader distribution. However, the distribution was asymmetric with very few data points below 10% PARP1-positive area and a long tail with up to 40% PARP1-positive tissue area (Fig. 1e).

The performance of PARP1 as a classifier for tumor and normal tissue was evaluated using a receiver operating characteristic (ROC) curve (Supplementary Fig. S2b). For all samples, tumor tissue could be detected by PARP1 expression with a specificity of 0.972 and a sensitivity of 0.974. The positive predictive value (PPV) was 0.982 and the negative predictive value (NPV) was 0.958 for correct classification of tumor and normal tissue, when the threshold between malignant and normal was set at 6.5% PARP1-positive area. We further determined the probability of a tissue sample to be malignant based on its PARP1-positive area and found that the probability of a given tissue area to be tumor increased from 0% to 100% between 5% and 9% PARP1-positive area (Supplementary Fig. S2c).

### PARP1 protein expression in OSCC xenografts

**Figure 2:**
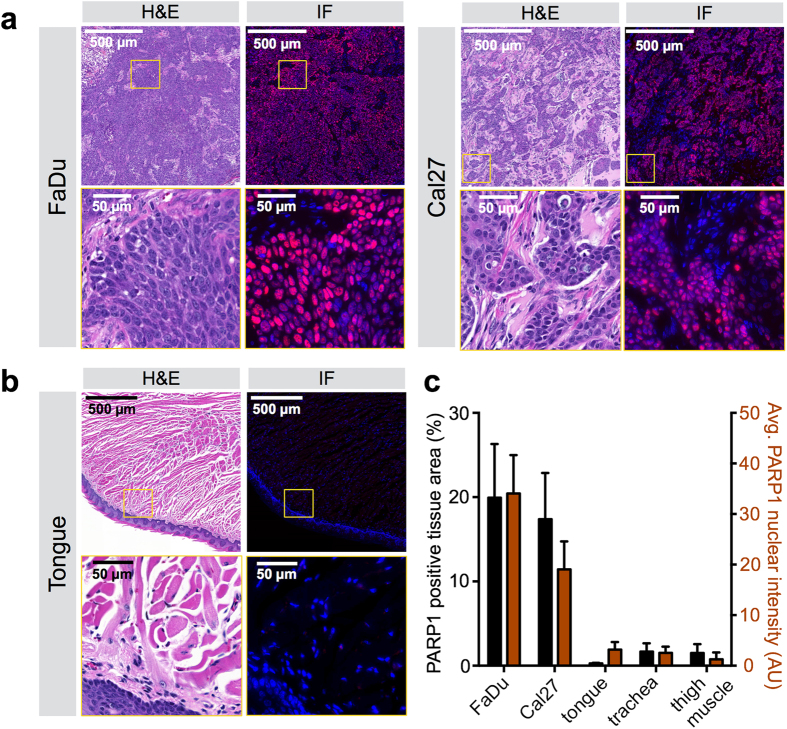
Immunofluorescence staining of PARP1 in human oral cancer squamous cell carcinoma xenografts (OSCC). (**a**) H&E staining with corresponding PARP1 immunofluorescence microscopy of OSCC xenografts. (**b**) H&E staining with corresponding PARP1 immunofluorescence microscopy of normal mouse tongue. Blue = cell nuclei stained with DAPI; red = PARP1 staining. (**c)** Quantification of the PARP1-positive area in relation to the whole tissue area and average intensity of PARP1 staining in all nuclei, for various xenograft and normal mouse control tissues. The PARP1-positive tissue area was determined by performing a thresholding on red (PARP1) and green (autofluorescence of total tissue, not displayed here) areas and the relative PARP1-positive area was calculated by dividing the PARP1-positive area by the total tissue area. Values display means ± SD of n = 10 values per tumor/organ. See Supplementary Fig. S3 for images of trachea and thigh muscle, as well as images obtained with a staining control.

Expression of PARP1 was found to be similar in two xenograft models of human OSCC (Fig. 2a,b). The two OSCC cell lines FaDu and Cal27 contained PARP1 in 19.9 ± 6.4% (FaDu) and 17.4 ± 5.5% (Cal27) of the tissue area and displayed a PARP1 immunofluorescence intensity of 34.1 ± 7.6 AU (FaDu) versus 19.1 ± 5.5 AU (Cal27), respectively. These values were highly elevated compared to normal mouse tongue, trachea, and muscle, in which the PARP1-positive area was below 2% and the maximum intensity was 1.2 AU and 3.2 AU for muscle and tongue, respectively (Fig. 2c). PARP1 immunofluorescence staining was only positive in the nuclei of tumor cells, but not stromal cells or cytoplasmic areas of cancer cells (Supplementary Fig. S3a), as described previously^15^. Furthermore, we showed that negligible non-specific staining was observed when the anti-PARP1 antibody was replaced with a non-specific rabbit IgG isotype control (Supplementary Fig. S3b).

### *Ex vivo* PARP1 imaging with PARPi-FL in subcutaneous OSCC xenografts

**Figure 3:**
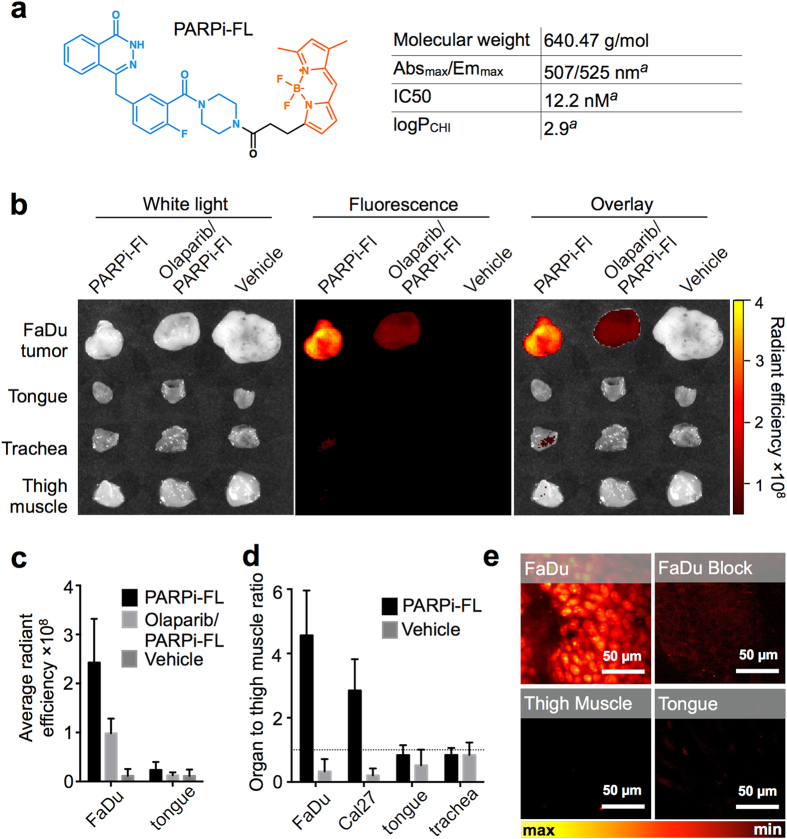
PARPi-FL accumulation in OSCC xenografts in mice. (**a**) Molecular structure^25^,^48^ and physicochemical properties of PARPi-FL (blue = DNA binding domain; orange = BODIPY^®^ FL fluorescence dye moiety); ^a^Irwin *et al.* 2014^43^. (**b**) Representative epifluorescence images of FaDu tumor, tongue, muscle, and trachea. Radiant efficiency displayed in units of [(photons/s/cm^2^/sr)/(μW/cm^2^)]. (**c**) Average radiant efficiency of FaDu tumors and tongue. (**d**) Tumor/organ-to-muscle ratios from images of FaDu and Cal27 tumors, tongue, and trachea calculated as the average radiant efficiency in a region of interest. (**c**,**d**) display means and SD from n ≥ 5 tissue specimens for PARPi-FL, Olaparib/PARPi-FL, and n ≥ 3 for vehicle control. Images and semiquantitative analyses of fluorescence intensities were acquired *ex vivo* 90 minutes post-injection of PARPi-FL (75 nmol/167 μl PBS with 30% PEG300), Block (Olaparib; 3750 nmol/100 μl PBS with 30% PEG300 30 minutes prior to PARPi-FL), or vehicle (167 μl PBS with 30% PEG300). (**e**) Confocal images of PARPi-FL signal in freshly excised tumor, tongue, and trachea at 90 minutes post-injection of PARPi-FL or Block (as described in (**a**)).

Next, we determined whether FaDu and Cal27 tumors accumulated PARPi-FL after intravenous injection, and if tumor uptake was due to binding to PARP1. PARPi-FL is a targeted imaging agent that fluoresces in the visible range (Fig. 3a) and accumulates in the nuclei of PARP1-expressing cells^25^. Epifluorescence imaging of excised subcutaneous FaDu and Cal27 tumors was performed 90 minutes after injection of PARPi-FL (75 nmol PARPi-FL, 0.5 mM, in PBS with 30% PEG300), and the intensity of the fluorescence signal was compared to normal tongue, trachea, and control thigh muscle tissue. PARPi-FL generated a strong fluorescence signal in tumors and almost no fluorescence in normal tissues (Fig. 3b). We confirmed the specificity of the signal by injecting the non-fluorescent PARP1-targeted drug Olaparib before administration of PARPi-FL, which resulted in a reduction of the fluorescent signal of the tumor by 60% (average radiant efficiency PARPi-FL: 2.4 × 10^8^ versus Olaparib/PARPi-FL: 0.98 × 10^8^, P < 0.001, Fig. 3c). The signal in the tongue was low, independent of the blocking (average radiant efficiency PARPi-FL: 0.24 × 10^8^ versus Olaparib/PARPi-FL: 0.13 × 10^8^, P = 0.3, Fig. 3c), further supporting that there is little expression of PARP1 in tongue tissue, and that little of the imaging agent is nonspecifically bound. In control mice injected with vehicle only, fluorescence signals did not exceed an average radiant efficiency of 0.11 × 10^8^ in either tumor or tongue.

We quantitatively evaluated the fluorescence signal by tissue-to-thigh-muscle ratios. This ratio was 4.6 ± 1.4 for FaDu tumors and 2.9 ± 1.0 for Cal27 tumors (Fig. 3d). In mouse tongues and trachea, which represent the surrounding healthy tissues in oral cancer, the fluorescence signals were not elevated compared to thigh muscle (uptake ratio: 0.8 ± 0.3 for tongue, and 0.3 ± 0.2 for trachea). Microscopic analysis of the fluorescence distribution in freshly excised tissue further confirmed the specific accumulation of PARPi-FL in tumor cells, since a strong nuclear fluorescence was only observed in FaDu tumors, but not in tongue or muscle after PARPi-FL injection (Fig. 3e).

### Correlation of PARP1 expression on IHC and PARPi-FL localization

**Figure 4:**
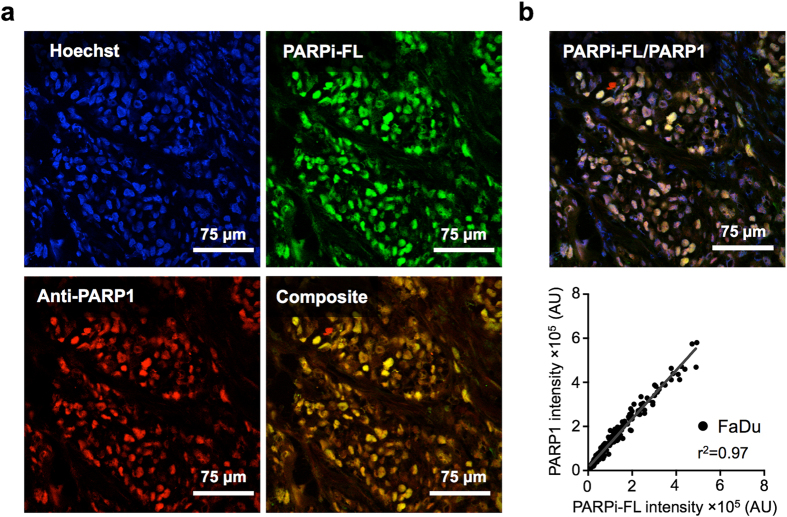
Localization of PARPi-FL in relation to cell nuclei and PARP1 protein using confocal imaging. (**a**) Images are shown of cell nuclei that were stained (*ex vivo*) with Hoechst 33342 (blue), fluorescence from PARPi-FL injected intravenously *in vivo* (green), and PARP1 that was stained with an anti-PARP1 antibody (*ex vivo*) and detected via immunofluorescence (red). (**b**) Correlation analysis (Pearson R squared) between intravenously injected PARPi-FL and PARP1 immunofluorescence in FaDu tumor tissue, based on the intensity of green or red fluorescence in the nuclear area. The nuclear area was determined via a threshold based on blue fluorescence (Hoechst). A total of 222 correlation pairs were pooled from n = 3 tumor sections.

PARP1 antibody staining was highly co-localized with PARPi-FL fluorescence (R_coloc._ = 0.986, R^2^ = 0.973; 95% confidence interval 0.98 to 0.989; Fig. 4a,b). This suggests that PARPi-FL not only binds specifically to PARP1-expressing cells but also quantitatively reflects the amount of PARP1 present in a cell (Fig. 4b). There was no bleed-through/cross-contamination of PARPi-FL into the PARP1 channel and vice versa, shown by PARP1 and PARPi-FL control experiments (Supplementary Fig. S4).

### PARPi-FL optical imaging of orthotopic OSCC

**Figure 5:**
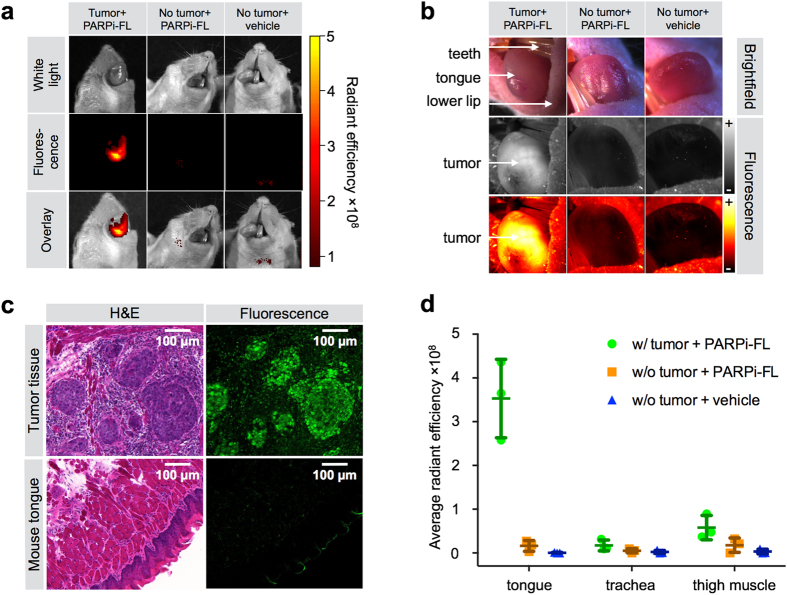
Detection of an orthotopic OSCC xenograft model in mouse tongue. (**a**) Epifluorescence imaging of tumor-bearing tongues (FaDu) and healthy mouse tongues from animals injected with PARPi-FL (75 nmol/167 μl PBS with 30% PEG300) or vehicle (PBS with 30% PEG300) 90 minutes prior to imaging. (**b**) Imaging with a Lumar fluorescence stereoscope. Images were taken in brightfield and with 488 nm laser excitation. Fluorescent images are displayed in grayscale and intensity-scaled (“+” = maximum signal, “−” = minimum signal). (**c**) Cryopreserved tongue sections were imaged with a confocal microscope to detect PARPi-FL following fixation. H&E staining in adjacent sections for anatomical and pathological evaluation of PARPi-FL localization. (**d**) Average radiant efficiency [p/s/cm^2^/sr]/[μW/cm^2^] of PARPi-FL in excised tumor-bearing and healthy tongues as well as trachea and thigh muscle. Means and SD from n = 3 animals/group are displayed.

PARPi-FL uptake was also imaged *in vivo* in an orthotopic tongue tumor model of OSCC (FaDu cells) using the same parameters as for subcutaneous tumor imaging *ex vivo* (intravenous injection of 75 nmol PARPi-FL/animal, imaging 90 minutes post-injection). Here, epifluorescence imaging showed a strong PARPi-FL accumulation in parts of the tongue that were visibly affected by OSCC, whereas no signal accumulation was observed in tongues without tumors after PARPi-FL or vehicle injection (Fig. 5a). Further, PARPi-FL accumulation in tongue tumors was visualized using a fluorescence stereoscope. This imaging technique is closer to the clinical situation, where real-time fluorescence imaging is required. PARPi-FL accumulation in orthotopic tumors was clearly visible and confined to areas that were macroscopically identifiable as pathologic (Fig. 5b). Histological evaluation of tissue sections from tumor-bearing tongues confirmed that PARPi-FL could only be found in regions classified as tumor tissue in H&E stained sections (Fig. 5c), and that the tumors showed highly elevated PARP1 expression compared to normal mouse tongue (Supplementary Fig. S5). *Ex vivo* signal quantification in excised orthotopic and healthy tongues, trachea, and muscle revealed a 6.1-fold higher radiant efficiency in orthotopic tumors than in thigh muscle (3.5 ± 0.9 × 10^8^ and 0.6 ± 0.3 × 10^8^ for orthotopic tumors and thigh muscle, respectively; P < 0.01). When no tumor was present tongue and thigh muscle showed the same fluorescence signal (radiant efficiency 0.2 ± 0.1 × 10^8^ and 0.2 ± 0.2 × 10^8^, respectively; P = 0.1) (Fig. 5d).

### Cellular resolution imaging of PARPi-FL in whole tumors

**Figure 6:**
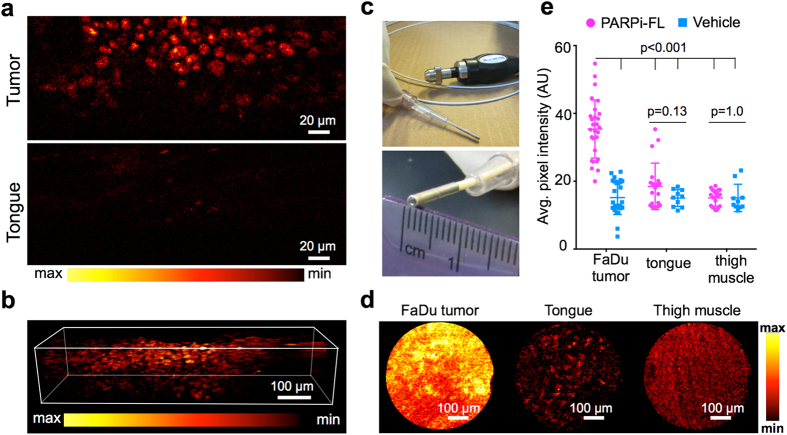
Microscopic imaging of whole excised FaDu tumors. (**a**) Whole excised FaDu tumor and mouse tongue were imaged 90 minutes post-injection of PARPi-FL (75 nmol/167 μl PBS with 30% PEG300) using a custom-built dual-axis confocal microscope with 488-nm laser excitation. (**b**) Reconstruction of a Z-Stack spanning a depth of 250 μm to show PARPi-FL nuclear localization. (**c**) Whole excised FaDu tumors were also imaged using a commercial confocal laser endomicroscope featuring a flexible microprobe with a resolution of 1.4 μm. (**d**) FaDu tumor, mouse tongue, and muscle were imaged 90 minutes post-injection of PARPi-FL (75 nmol/167 μl PBS with 30% PEG300) following sacrifice of the animals. Images are representative frames within real-time video recordings of the organs at 488-nm laser excitation. Identical window/leveling has been applied in all images. (**e**) Analysis of signal intensity after PARPi-FL or vehicle injection. Ten frames per specimen were analyzed for their average pixel intensity using ImageJ (n = 3). Statistical significance was determined using an unpaired t-test, corrected for multiple comparisons by the Holm-Šidák method with an alpha of 0.05.

To show that PARPi-FL is suitable for imaging of tumors at cellular resolution, we conducted imaging of freshly excised FaDu tumor tissue 90 minutes after injection of PARPi-FL using a custom dual-axis confocal microscope at a range of depths (Supplementary Fig. S6a and Fig. 6a,b) and a commercial fluorescence endomicroscope at a fixed depth (Fig. 6c,d). This instrument enabled the identification of single cells based on their nuclear PARPi-FL uptake at up to 200 μm below the tissue surface, whereas negligible signal was detected in normal tongue tissue from animals injected with PARPi-FL (Fig. 6b and Supplementary Fig. S6b). The dual-axis confocal microscopy technique can be developed into a hand-held device, which could then be translated into clinical practice in the future^26^,^27^. Fluorescence endomicroscopy, a technique that has already been implemented in clinical practice^12^,^28^,^29^, was also able to clearly distinguish between FaDu tumors and control tissues (Fig. 6c). When no PARPi-FL was injected, no difference in fluorescence intensity of FaDu tumors, tongue, or muscle tissue was observed (Supplementary Fig. S7). When compared to the vehicle control group, the average signal intensity of FaDu tumors was significantly increased 90 minutes after PARPi-FL injection (35.4 ± 8.6 AU and 15.2 ± 5.0 AU, respectively; P < 0.001). There was no difference between the average signal intensity after PARPi-FL or vehicle injection in tongue and thigh muscle (18.5 ± 6.9 AU and 15.0 ± 2.5 AU for tongue, with and without PARPi-FL administration, respectively, P = 0.13; 15.1 ± 2.4 AU and 15.1 ± 4.0 AU for thigh muscle, with and without PARPi-FL administration, respectively, P = 1.0; Fig. 6e).

### Oral cancer imaging after topical application of PARPi-FL

**Figure 7:**
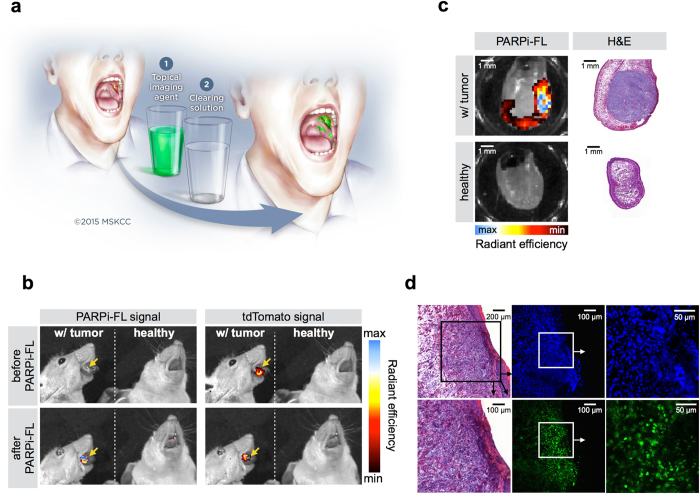
Oral cancer delineation after topical application of PARPi-FL. (**a**) Possible imaging procedure for OSCC detection using a PARPi-FL based, orally applied solution. Imaging of the oral cavity with a fluorescence camera will be conducted after a one minute topical PARPi-FL application, followed by removal of unbound or non-specifically bound compound with the clearing solution 1% acetic acid. The illustration is courtesy of MSK. (**b**) Spectrally resolved epifluorescence imaging of healthy mice and tongue tumor bearing mice before and after topical application of PARPi-FL. The tdTomato signal indicates the position of the tumor, while the PARPi-FL signal shows the ability of PARPi-FL to specifically detect sites of OSCC after topical application. All images are scaled to the same maximum radiant efficiency. (**c**) Epifluorescence imaging of healthy mice and mice bearing orthotopic tongue tumors. The tumors express tdTomato fluorescent protein, as control for tumor localization. Imaging of PARPi-FL signal was conducted before and after PARPi-FL topical application. (**d**) Confocal microscopy of an OSCC bearing tongue after topical application of PARPi-FL *in vivo*. Green: PARPi-FL, blue: Hoechst DNA stain. H&E confirms presence of tumor. Arrows point to enlarged images of the squared area.

Topical application of a PARPi-FL formulation (30% PEG300/PBS) with subsequent fluorescence screening of the oral cavity for OSCC detection could improve the current standard of care, particularly in low resource settings (Fig. 7a)^8^. In a preclinical model of tongue OSCC, we investigated if PARPi-FL tumor contrast after topical application was comparable to intravenous injection. Macroscopic evaluation using epifluorescence imaging confirmed that PARPi-FL colocalized to areas of the tongue where there was the orthotopic tumor, as confirmed by tumor cells expressing the fluorescent protein tdTomato (Fig. 7b). Correlation of PARPi-FL signal with H&E staining of tongue sections confirmed that the agent’s retention after topical application is strongest in regions of tumor growth, but only if it is close to the surface of tongue (Fig. 7c). Microscopic evaluation revealed that PARPi-FL was able to, penetrate up to 300 μm deep into tumor tissue during the one minute application window, whilst being able to wash out from non-target areas during the cleaning steps, resulting in high contrast PARP1 staining of OSCC nuclei (Fig. 7d).

## Discussion

Our results indicate that PARP1 protein expression is markedly increased in OSCC when compared to normal tissues of the oral cavity. Moreover, we demonstrate that the small molecular imaging agent PARPi-FL can be used to delineate OSCC in living mice. PARPi-FL is efficiently retained in oral cancer tissue, yielding a strong imaging signal, paired with high contrast to surrounding normal tissue. This enabled high-resolution *in vivo* imaging of orthotopic OSCC with clinically translatable instruments. Using these devices, PARP1 expression was imaged from the macroscopic to the subcellular level. We were able to show that PARPi-FL, due to its high tissue permeability (4.7 ± 2.5 μm/s)^30^, efficiently penetrates into tumor tissue after topical application, and selectively accumulates in OSCC cells close to the tissue surface, while being washed out from non-target tissues and compartments within minutes.

Our findings regarding PARP1 expression in human oral cancer are consistent with previously published research in which PARP1 overexpression was also found in other types of cancer, including breast cancer^17^,^23^,^31^,^32^,^33^, colorectal cancer^19^,^34^,^35^, prostate cancer^21^, and glioblastoma^20^. PARP1 expression was elevated throughout the patient-derived OSCC samples. And PARP1 expression per nucleus was fairly uniform^25^. However, the density of PARP1-positive tumor cells varies in different areas. Specifically, PARP1 expression levels were higher at the invasive margins of the tumors than in the center. The impact of tumor cell density is also apparent in Fig. 1d, where T2 specimens have higher PARP1 expression than the more necrotic T3 and T4 specimens. Interestingly, the premalignant specimens in our study showed equally high PARP1 expression levels as malignant specimens. Premalignant tissues, such as severe dysplasia or carcinoma *in situ*, have been shown to be associated with progression to cancer^36^,^37^. The overexpression of PARP1 in premalignant tumors is promising for early diagnosis of OSCC and could become interesting for therapeutic applications as well.

The sample size for the statistical analysis was relatively small but we were able to find statistically significant differences. To ensure that are findings are unlikely to be false positive we have used stringent multiple comparison methods. But we are cognizant of the fact that our analysis lacked power and hence may have missed other significant findings.

PARP1 is an attractive target for tumor detection because its increased expression in a large number of cancers. Other members of the PARP family, such as PARP2, which is also inhibited by Olaparib, is less abundant and its expression was found not to be upregulated in a number of primary cancers^23^. Although, to our knowledge, no data on PARP2 expression in oral cancer are available, this is pointing towards a less important role of PARP2 in tumorigenesis and a low suitability as cancer imaging agent compared to PARP1^33^. PARP1 overexpression is believed to be due to the increased DNA damage occurring in genetically unstable cancer cells, rather than the activation of specific oncogenic pathways^23^. Furthermore, the density of nuclei is typically higher in malignant tumors than in most normal tissues^38^. The PARPi-FL *in vivo* imaging signal therefore reflects both the higher expression levels of PARP1 per nucleus as well as the higher nuclear density in malignant tumors. Thus, PARPi-FL could potentially be used to image a large variety of tumors during screening or surgery. OSCC is an obvious candidate for the initial evaluation of PARPi-FL imaging because of the clinical needs for better detection and delineation of OSCC, as well as its easy accessibility for fluorescence imaging.

In the field of optical fluorescence imaging, a large number of probes absorb and emit near-infrared light. In this wavelength range (650–900 nm)^39^, photons are less scattered and absorbed by tissues, which allows for better tissue penetration. In addition, there is less background autofluorescence from tissues with near-infrared excitation, as compared to visible excitation^40^,^41^. The BODIPY^®^ FL fluorophore used to synthesize PARPi-FL operates in the visible range of light (400–700 nm), but it has the added advantage of an exceptionally low molecular weight and it does not ionize under physiological conditions. These properties allow for efficient *in vivo* extravasation, combined with fast cell permeation and intranuclear accumulation. Larger fluorophores and charged molecules result in significantly reduced cell permeability, together with low contrast ratios^30^.

Our *in vivo* imaging results confirm that the fluorescence from BODIPY^®^-FL allows for high-contrast imaging of superficial tumors in the tongue despite the known limitations of green fluorescent dyes. Moreover, fluorescence imaging systems that operate with a green fluorescence channel have entered clinical practice, e.g., probe-based confocal laser endomicroscopy (pCLE), which is FDA-approved for imaging the entire gastrointestinal tract, including the oral cavity. The utility of pCLE imaging for better differentiation of nondysplastic, precancerous, and cancerous lesions of the head and neck in patients has already been shown using fluorescein, a nonspecific green fluorescent dye^42^, which absorbs and emits very closely to PARPi-FL (Excitation/Emission max.; fluorescein: 490/525 nm; PARPi-FL: 507/525 nm^43^). We were able to image PARPi-FL accumulation in OSCC with high contrast using a clinically approved pCLE system.

Prospectively, and envisioning a PARPi-FL assisted oral cancer screening procedure, the application method of PARPi-FL should be switched from intravenous to topical application, reducing complexity, and increasing the agent’s breadth and versatility in the clinic. Topical application would further reduce cost, potential side effects and streamline the imaging protocol. Optical fluorescence imaging equipment is lower priced and has a higher grade of mobility compared to other molecular imaging modalities, for example PET or MRI.

We were able to show that PARPi-FL penetrates up to 300 μm into tissue, sufficient for detection of OSCC, a disease that typically originates within the outermost cell layers of the oral cavity^5^,^44^.

In conclusion, our results indicate that PARPi-FL imaging of OSCC is very promising for a variety of applications, including cancer screening, surgical guidance during tumor removal, and delineation of tumor margins by pCLE. Hence, PARP1 imaging could result in earlier detection of oral cancer and reduce the morbidity of radical surgery that plagues patients suffering from OSCC.

## Materials and Methods

### Cell culture

The OSCC cell lines FaDu (hypopharyngeal SCC; ATCC, Manassas, VA) and Cal27 (tongue SCC; ATCC, Manassas, VA) were grown in a monolayer culture at 37 °C in a 5% CO_2_ humidified atmosphere. FaDu cells were maintained in MEM medium and Cal27 cells in D-MEM medium, both containing 10% (v/v) FBS and 1% PenStrep.

### Animal models

Female athymic nude mice (NCr-Foxn1nu, Taconic, Hudson, NY) were housed under standard conditions with water and food ad libitum. Throughout all procedures, animals were anesthetized with 2% isoflurane. To implement subcutaneous human OSCC tumors, 2 × 10^6^ FaDu or Cal27 cells were dispensed in 100 μl of a 1/1 mixture of medium/Matrigel™ (BD Biosciences, Bedford, MA) and were injected into the lower back of the animals. Experiments were conducted when tumors reached 100–150 mm^3^ volume. For an orthotopic OSCC model, 5 × 10^5^ FaDu or FaDu^tdTomato^ (FaDu stably transfected with tdTomato fluorescent protein; Creative Biogene, Shirley, NY) cells in 20 μl PBS were injected directly into the tongue and the mice were observed daily for tumor growth and weight loss. Imaging was conducted usually after 3–4 weeks. All animal experiments were performed in accordance with institutional guidelines and approved by the IACUC of MSK, and followed NIH guidelines for animal welfare.

### Human tissues

Quantification of PARP1 expression was carried out using human tongue tumor specimens (n = 12), obtained from the Department of Pathology of MSK. The use of tissues was approved by the Institutional Review Board (IRB) at MSK and informed consent was obtained from all subjects.

### PARP1 expression in tissues

PARP1 antigen in human oral cancer tissue, as well as FaDu and Cal27 xenografts and mouse tissues was detected using immunohistochemical (IHC) and immunofluorescence (IF) staining techniques, which were performed at the Molecular Cytology Core Facility of MSK using the Discovery XT processor (Ventana Medical Systems, Tucson, AZ). The anti-PARP1 rabbit polyclonal antibody (sc-7150, Santa Cruz Biotechnology, Santa Cruz, CA) specifically bound both human and mouse PARP1 (0.2 μg/ml). Paraffin-embedded formalin fixed 3 μm sections were deparaffinized with EZPrep buffer, antigen retrieval was performed with CC1 buffer (both Ventana Medical Systems, Tucson, AZ), and sections were blocked for 30 minutes with Background Buster solution (Innovex, Richmond, CA). Anti-PARP1 antibody was incubated for 5 hours, followed by 1 hour of incubation with biotinylated goat anti-rabbit IgG (PK6106, Vector Labs, Burlingame, CA) at a 1:200 dilution. For IHC detection, a DAB detection kit (Ventana Medical Systems, Tucson, AZ) was used according to the manufacturer’s instructions, sections were counterstained with hematoxylin and coverslipped with Permount (Fisher Scientific, Pittsburgh, PA). IF detection was performed with Streptavidin-HRP D (from DABMap Kit, Ventana Medical Systems), followed by incubation with Tyramide Alexa Fluor 594 (T20935, Invitrogen, Carlsbad, CA) prepared according to the manufacturer’s instructions. Sections were counterstained with 4′,6-diamidino-2-phenylindole (DAPI) for 10 minutes and coverslipped with Mowiol^®^ mounting medium (Sigma-Aldrich, St. Louis, MO). Incubating with a rabbit IgG instead of the primary antibody controlled for non-specific binding of the secondary antibody. For morphological evaluation of tissue characteristics, H&E staining was performed on adjacent sections.

### Quantification of PARP1 expression

For PARP1 protein quantification, stained tumor sections were digitalized using a MIRAX Slide Scanner (3DHISTECH, Budapest, Hungary). On at least 10 fields of view per section, PARP1 presence was quantified using MetaMorph^®^ Software (Molecular Devices, Sunnyvale, CA). In IHC stained tissues, a thresholding was performed on brown (PARP1) and blue (tissue) areas and the relative PARP1-positive area was calculated by dividing the brown area by the total tissue area. For IF, the PARP1-positive area was determined by thresholding the red fluorescent area and dividing it by the whole tissue area, which was determined based on autofluorescence in the green channel. PARP1 intensity was also determined by measuring the red fluorescence intensity in all nuclei, which were thresholded using DAPI staining. The measured fluorescence intensities were averaged over all nuclei in each field of view, with intensity values ranging from 0–255.

### Synthesis of PARPi-FL

Synthesis of the optical imaging agent PARPi-FL was carried out as previously described^25^. In summary, the green fluorescent dye BODIPY-FL NHS-ester (Invitrogen, Carlsbad, CA) was conjugated to 4-(4-fluoro-3-(piperazine-1-carbonyl)benzyl)phthalazin-1(2H)-one and purification by preparative HPLC (Waters’ XTerra C-18 5 μm column, 7 ml/min, 5% to 95% of acetonitrile in 15 min) afforded PARPi-FL in 70–79% yield as a red solid. Analytical HPLC analysis (Waters’ Atlantis^®^ T3 C18 5 μm 4.6 × 250 mm column) showed high purity (>97%) of the imaging agent. The identity of PARPi-FL was confirmed using ESI-MS (MS(+) m/z = 663.4 [M + Na]^+^). For imaging studies, PBS (117 μl) was slowly added to an aliquot of PARPi-FL (50 μg, 75 nmol) in 50 μl of poly(ethylene glycol) (PEG300, Sigma-Aldrich, St. Louis, MO) to obtain a final injection volume of 167 μl.

### Imaging of PARPi-FL uptake in subcutaneous human OSCC xenografts

For evaluation of the uptake of PARPi-FL in subcutaneous OSCC xenografts, animals carrying either FaDu or Cal27 tumors were intravenously injected with PARPi-FL (75 nmol/167 μl PBS with 30% PEG300 (Sigma-Aldrich, St. Louis, MO)) (n ≥ 6/group). To assess the specificity of PARPi-FL accumulation in one group of animals, we injected a 50-fold excess (3.75 μmol/100 μL PBS with 30% PEG300) of Olaparib (LC Laboratories, Woburn, MA) 30 minutes prior to the PARPi-FL injection, blocking the specific binding sites in FaDu tumors (n = 4). Animals were sacrificed 90 minutes post-injection and tumors, tongues, trachea, and muscle were excised and imaged using epifluorescence imaging (IVIS Spectrum, PerkinElmer, Waltham, MA). For detection of the fluorescent PARPi-FL emission, we used the predefined GFP Filterset (excitation: 465/30 nm, emission: 520-580 nm) and subsequently removed autofluorescence through spectral unmixing. Semiquantitative analysis of the PARPi-FL signal was conducted by measuring the average radiant efficiency in regions of interest (ROIs) that were placed on all organs under white light guidance. This measure carries the unit [p/s/cm^2^/sr]/[μW/cm^2^] and is defined as the number of photons per second leaving a square centimeter of tissue and radiating into a solid angle of one steradian (sr). Resulting numbers are normalized for the integration time, binning, f/stop, field of view, illumination intensity, and the ROI area, making measurements comparable among each other. Freshly excised whole tumors were also microscopically imaged directly after epifluorescence imaging; tissues were placed on a cover slip with a freshly cut surface facing the cover slip and images were taken on an inverted laser scanning confocal microscope using 488 nm laser excitation (LSM 5-Live, Zeiss, Jena, Germany).

### Correlation of PARPi-FL uptake and PARP1 expression

To determine the specificity of the accumulation of PARPi-FL within tumor tissue, the inter- and intracellular co-localization of the targeted fluorescent probe with PARP1 antigen was determined in histological sections. FaDu xenografts and control tissues (tongue, muscle) were snap-frozen 90 minutes after intravenous injection of PARPi-FL (75 nmol/167 μl 30% PEG300 in PBS). Next, 10 μm cryosections were fixed in 4% paraformaldehyde for 8 minutes, followed by blocking with 3% (v/v) goat serum (Sigma-Aldrich, St. Louis, MO) in PBS. Antibodies were diluted in 1% (w/v) BSA and 0.3% (v/v) Triton X-100 in PBS. Anti-PARP1 primary antibody (sc-7150, Santa Cruz Biotechnology, Santa Cruz, CA) was incubated overnight at 4 °C (1 μg/ml), followed by three 10-minute washes with PBS and incubation with secondary AlexaFluor^®^ 680 goat anti-rabbit antibody (A21076, Molecular Probes, Eugene, OR) for 1 hour at 4 °C (2 μg/ml). After another 5-minute PBS wash, sections were mounted with Mowiol^®^ (Sigma-Aldrich, St. Louis, MO) containing Hoechst 33342 DNA Stain (Sigma-Aldrich, St. Louis, MO). Fluorescence images were captured using a Leica (Buffalo Grove, IL) SP8-inverse confocal microscope equipped with a 405 nm laser for detection of cell nuclei, a 488 nm laser for detection of *in vivo* applied PARPi-FL, and a 670 nm laser for detection of PARP1 antibody stain, each paired with suitable emission filters. Incubating sections with either a nonspecific rabbit IgG or PBS instead of primary antibody confirmed binding specificity. Bleed-through of signals into other channels was excluded by imaging sections that were either not injected with PARPi-FL *in vivo* (no signal should be seen in the 488 nm channel) or not stained with PARP1 (no signal should be seen in the 670 nm channel). Correlation analysis between PARPi-FL and PARP1 signal intensity was performed using MetaMorph^®^ Software (Molecular Devices, Sunnyvale, CA).

### Imaging of PARPi-FL uptake in orthotopic human OSCC xenografts

For orthotopic FaDu tongue tumors, epifluorescence imaging was conducted using the same procedure as described above, but animals were alive when imaged 90 minutes post-injection. All animals were anesthetized with 2% isoflurane in medical air. The tongues of all animals were exposed by opening their mouths and moving the tongue past the front teeth into the field of view of the IVIS. Animals were divided into three groups: tumor-bearing animals that were injected with PARPi-FL (75 nmol/167 μl PBS with 30% PEG300), healthy animals that were injected with PARPi-FL, and healthy animals that were injected with vehicle (167 μl PBS with 30% PEG300) (n = 3/group). Afterwards, animals were sacrificed and tongues, trachea, and thigh muscle were imaged *ex vivo*. Using the same experimental setup, imaging with a fluorescence stereoscope was conducted to show that the PARPi-FL signal could also be detected under real-time imaging conditions, as would be the case in the clinical setting. Here, the tongues of anaesthetized animals were imaged using 500/20 nm excitation and 535/30 emission filters and a fixed exposure time of 500 ms (SteREO Lumar.V12, Zeiss, Jena, Germany). Imaging was performed 90 minutes after intravenous injection of PARPi-FL (75 nmol/167 μl PBS with 30% PEG300).

### Imaging of OSCC xenografts using a dual-axis confocal microscope

To show the feasibility of intravital tumor imaging at cellular resolution, we imaged excised subcutaneous FaDu xenografts, tongue, and muscle 90 minutes after PARPi-FL (75 nmol/167 μl PBS with 30% PEG300) or vehicle injection using a custom-built dual-axis confocal microscope^45^. Illumination settings were optimal for BODIPY-FL imaging and settings (laser intensity and detector gains) were fixed for all tissues to ensure comparability (illumination intensity: 1.95–2.1 mW and photomultiplier gain setting: 0.656 V).

### Fluorescence endoscopy

We performed imaging of FaDu xenografts with a fluorescence endoscope that is available for both clinical and preclinical imaging (Cellvizio, Mauna Kea Technologies, Paris, France). It provides cellular to subcellular resolution and has a flexible confocal microprobe that enables versatile imaging. Here, after receiving a 90-minute post-injection of 150 nmol PARPi-FL (in 167 μl PBS with 30% PEG300) or vehicle (167 μl PBS with 30% PEG300), animals were sacrificed and skin was removed from subcutaneous FaDu tumors and thigh muscle (n = 4 PARPi-FL, n = 3 vehicle). The microprobe was slowly moved over the tumor, tongue, or muscle, while a real-time video was recorded using a 488 nm excitation beam. The videos were converted to grayscale and the intensity was measured in 10 frames per video using ImageJ 1.49e Software^3^.

### Topical application of PARPi-FL

For topical application of PARPi-FL, mice with or without orthotopic tongue tumors (FaDu^tdTomato^) were anaesthetized using ketamine (0.1 mg/g body weight) and tongues were exposed using forceps. For topical application, the tongues were dipped into a well of a 96-well plate filled with the respective incubation solution. The sequence of incubation was first 20 seconds in 1% acetic acid second 20 seconds PBS third 1 minute 5 μM PARPi-FL (30% PEG300/PBS) fourth 1 minute 1% acetic acid and fifth 10 seconds PBS. This was followed by cleaning of the tongue with an alcohol pad to remove residual unbound compound. The animals were imaged in the IVIS Spectrum before and after PARPi-FL application using the appropriate filter sets for detection of PARPi-FL and the tdTomato fluorescent protein. Spectral unmixing was used to separate the signals for tdTomato, PARPi-FL and autofluorescence. The tdTomato fluorescent protein allows *in vivo* localization of the tumor. For comparability, all images were scaled to the same maximum radiant efficiency. Imaging was repeated with sections of the excised tongues after cryofixation. Sections were fixated in 4% PFA, counterstained with Hoechst and imaged using a confocal microscope to localize PARPi-FL in the tissue. Adjacent sections were H&E stained for morphological evaluation.

### Statistical analysis

Statistical analysis of preclinical data was performed using GraphPad Prism 6 and R 3.1 (www.r-project.org). Unless otherwise stated, data points represent mean values, and error bars represent standard deviations of biological replicates. P values were calculated using a Student’s unpaired t-test, corrected for multiple comparisons by the Holm-Sidak method with an alpha of 0.05 as the cutoff for significance. For the clinical specimen, the distribution of the percent PARP1-positive area was separately estimated for normal and malignant tissues using kernel density estimation^46^. The ability to use PARP1 expression to distinguish malignant tissues from adjacent normal tissue was characterized by a receiver operating characteristic (ROC) curve. The probability of a given tissue being malignant as a function of the PARP1-positive tissue area (in percent) was estimated by nonparametric binary regression using the method of local likelihood^47^.

## Additional Information

**How to cite this article**: Kossatz, S. *et al.* Detection and delineation of oral cancer with a PARP1 targeted optical imaging agent. *Sci. Rep.* **6**, 21371; doi: 10.1038/srep21371 (2016).

## Supplementary Material

Supplementary Information
