# Supplementary material for: Detection and delineation of oral cancer with a PARP1 targeted optical imaging agent
Source: Sci Rep. 2016 Feb 22;6:21371. doi: 10.1038/srep21371 (PMC4761964; doi:10.1038/srep21371)
Supplement: Supplementary Information [file srep21371-s1.pdf]

Detection and delineation of oral cancer with a PARP1 targeted optical imaging agent

Authors: Susanne Kossatz[a], Christian Brand[a], Stanley Gutiontov[b], Jonathan T.C. Liu[c], Nancy Y. Lee[b], Mithat Gönen[d], Wolfgang A. Weber[a,e,f], Thomas Reiner[a,e*]

Affiliations:
[a] Department of Radiology, Memorial Sloan Kettering Cancer Center, New York, NY 10065, USA
[b] Department of Radiation Oncology, Memorial Sloan Kettering Cancer Center, New York, NY 10065, USA
[c] Department of Mechanical Engineering, University of Washington, Seattle, WA 98195, USA
[d] Department of Epidemiology and Biostatistics, Memorial Sloan Kettering Cancer Center, New York, NY 10065, USA
[e] Department of Radiology, Weill Cornell Medical College, New York, NY 10065, USA
[f] Molecular Pharmacology Program, Memorial Sloan Kettering Cancer Center, New York, NY 10065, USA

Correspondence:
*Thomas Reiner, Ph.D.
Department of Radiology
Memorial Sloan Kettering Cancer Center
1275 York Avenue
New York, NY 10065 USA
Phone: (646) 888-3461
reinert@mskcc.org

Keywords: PARP1, oral cancer, optical fluorescence imaging, targeted probes

Supplementary Fig. S1:

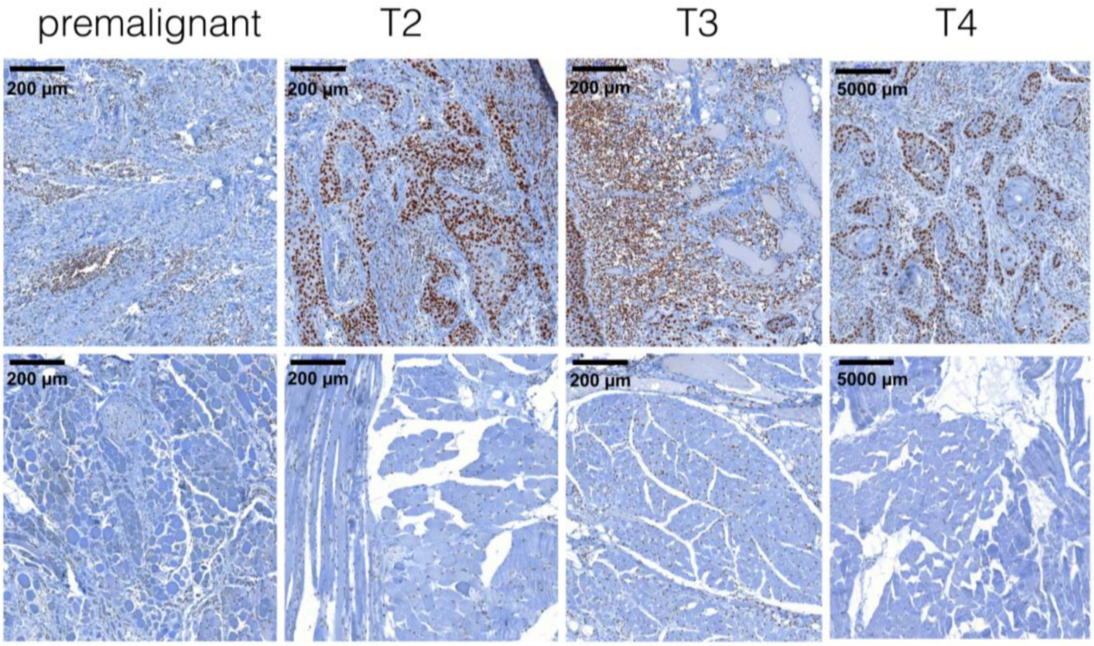

Fig S1. PARP1 staining of human oral cancer tissues. Representative PARP1 staining of different degrees of malignancy of human oral cancer tissue (upper row) and adjacent normal tongue tissue (lower row). PARP1 immunohistochemical staining was conducted on formalin-fixed, paraffin-embedded surgically removed tissues of human squamous cell carcinoma of the tongue using an anti-PARP antibody and IHC detection. Brown=PARP1-positive tissue. Blue=Hematoxylin counterstain.

Supplementary Fig. S2:

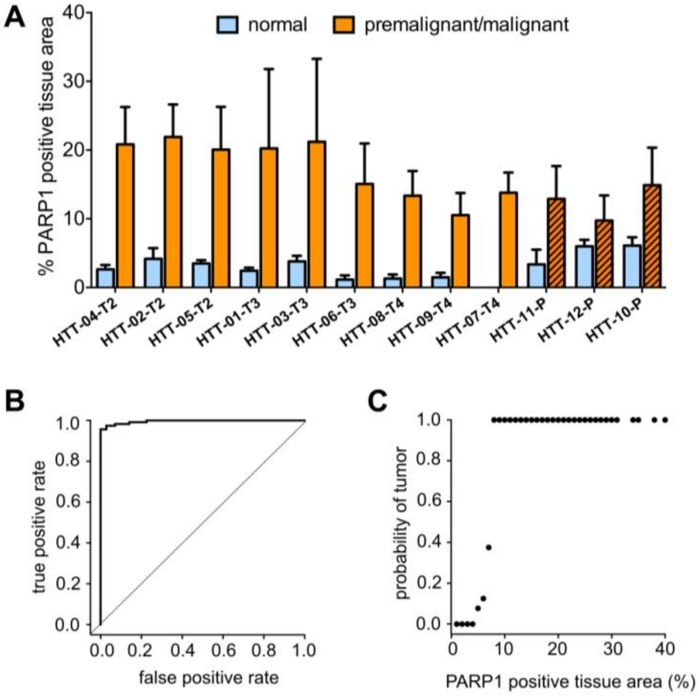

Fig. S2. PARP1 expression and statistics of human oral cancer tissues. (**A**) PARP1 quantification in human tongue tumor tissue displaying the PARP1-positive tissue area in %. For each specimen, malignant tissue and corresponding healthy adjacent tissue is shown. The PARP1-positive tissue area was determined as described in the methods section. For each sample, 5-10 data points in the tumor area or adjacent healthy tissue were analyzed. Displayed are means ± SD of all samples. HTT=human tongue tumor; color code: orange=malignant tissue (squamous cell carcinoma T2-T4), dashed orange=premalignant cases (moderate to severe dysplasia, carcinoma in situ), blue=corresponding normal adjacent tongue tissue. (**B**) The performance of PARP1 as a classifier for tumor and normal tissue was evaluated using a receiver operating characteristic (ROC) curve. (**C**) The probability of a given tissue being malignant as a function of the PARP1-positive tissue area (in percent) was estimated by nonparametric binary regression using the method of local likelihood.

Supplementary Fig. S3:

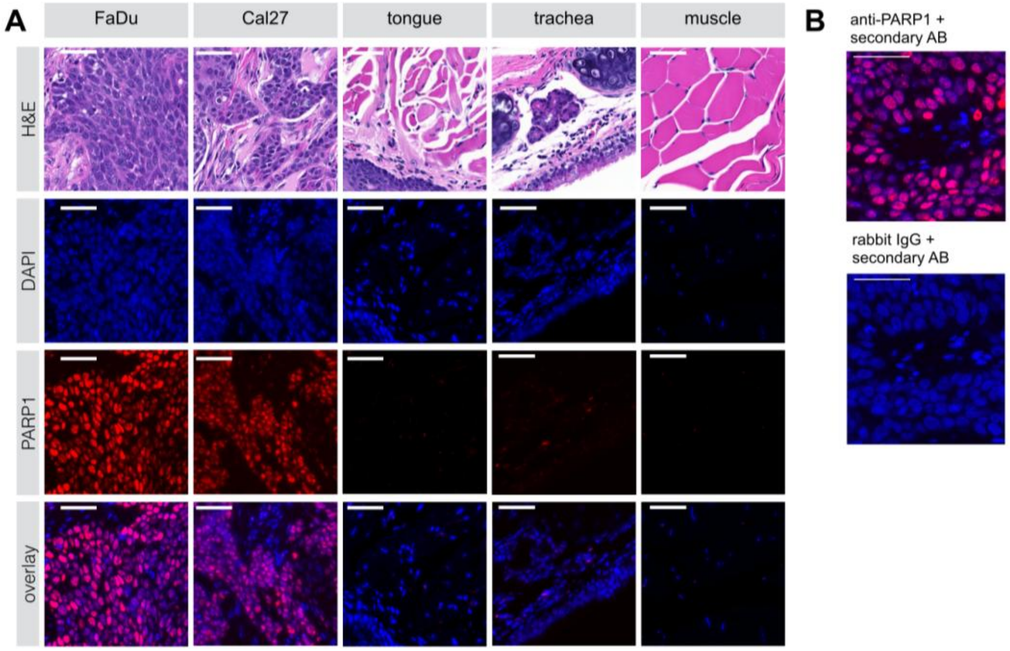

Fig. S3. PARP1 expression in subcutaneous mouse models of human oral cancer. (**A**) H&E staining with corresponding PARP1 IF staining of OSCC xenografts and mouse control tissues tongue, trachea, and muscle. Blue=cell nuclei stained with DAPI, red=PARP1 staining. Scale bar: 50 µM. (**B**) Control of staining specificity. The red PARP1 signal disappears when a nonspecific rabbit IgG is used instead of the anti-PARP1 primary antibody, showing the specificity of the secondary antibody for binding to the primary antibody. Scale bar: 50 µM.

Supplementary Fig. S4:

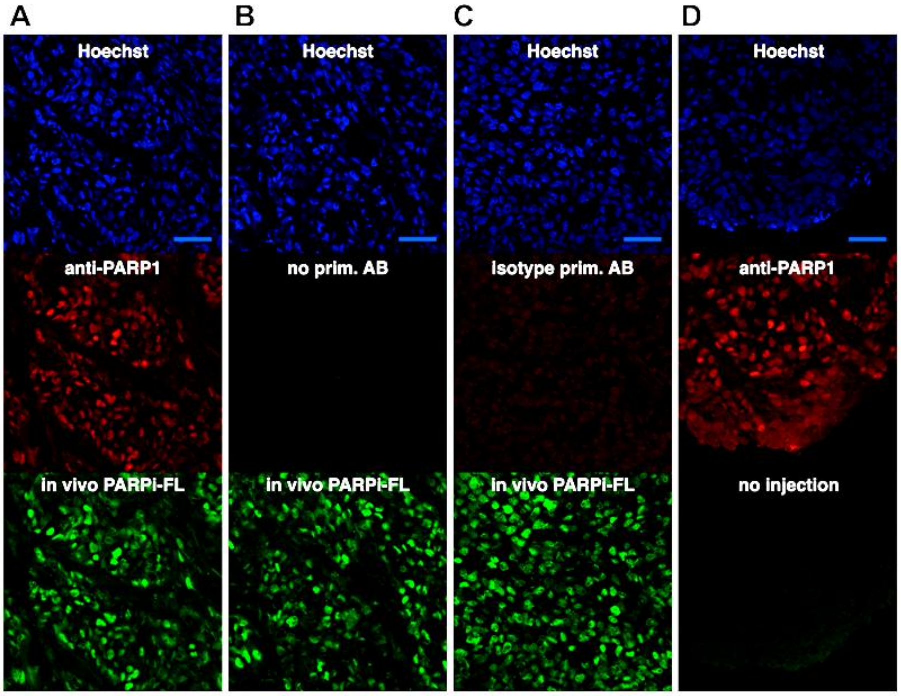

Fig. S4. Co-localization of PARP1 and PARPi-FL *in vivo*. Co-localization of PARP1 antibody staining with *in vivo* injected PARPi-FL in cryosections of FaDu xenografts. (**A**) Anti-PARP1 staining (red) co-localized with PARPi-FL (green) and both show a nuclear localization (blue; Hoechst staining). (**B**) No non-specific binding of the secondary antibody to the tissue occurred since the PARP1 signal (red) disappeared when PBS was used instead of the anti-PARP1 primary antibody. (**C**) When a rabbit IgG was used as isotype control instead of the anti-PARP1 antibody, only a very weak red staining was observed, showing minimal non-specific binding of the secondary antibody to the IgG. (**D**) When sections were only stained for PARP1, but no PARPi-FL was injected *in vivo*, there was no green nuclear signal, showing that no bleedthrough of signals through the fluorescence channels occurred. The images were taken on a Leica SP8 inverted confocal microscope. Scale bar: 50 μM.

Supplementary Fig. S5:

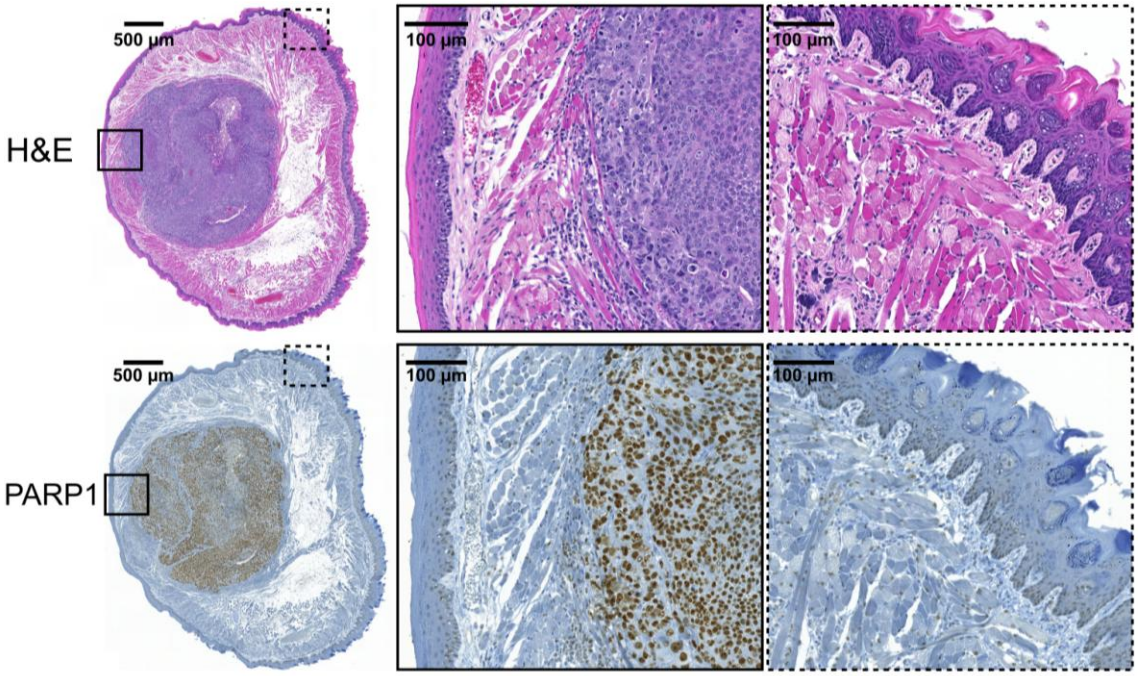

Fig. S5. PARP1 expression in an orthotopic mouse model of oral cancer. PARP1 staining in an orthotopic mouse model of oral cancer (derived from FaDu cells) with H&E staining of the corresponding areas. The PARP1 expression in the tumor area is much higher than in the surrounding normal tongue muscle and mucosal tissue. PARP1 immunohistochemical staining was conducted on formalin-fixed, paraffin-embedded tumor-bearing tongues of nude mice using an anti-PARP antibody and IHC detection. Brown=PARP1 positive tissue. Blue=Hematoxylin counterstain.

Supplementary Fig. S6:

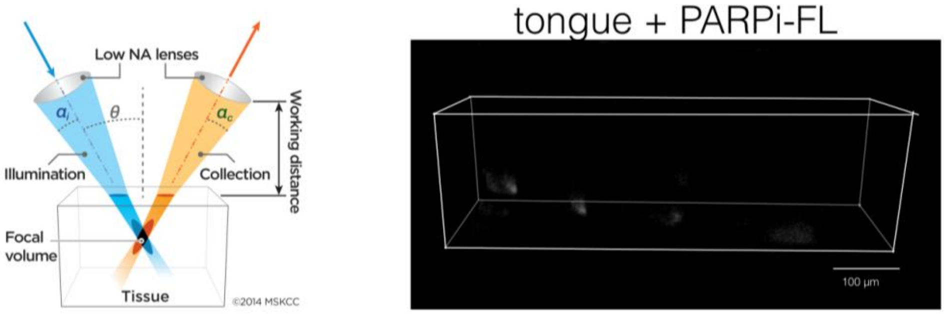

Fig. S6. Imaging of PARPi-FL accumulation using a dual-axis confocal microscope. (**A**) Working principle and beam paths of the custom-built dual-axis confocal microscope with 488-nm laser excitation. (**B**) Reconstruction of a Z-Stack of images of normal mouse tongue 90 minutes after PARPi-FL injection. No nuclear accumulation can be seen. The only visible signal is produced by the papillae on the tongue surface of mice.

Supplementary Fig. S7:

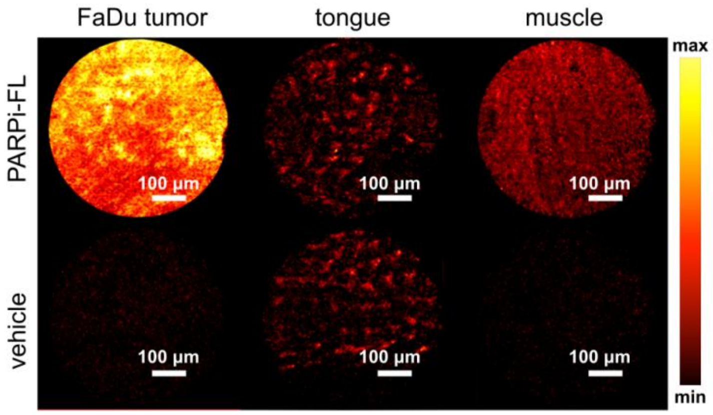

Fig. S7. Imaging of PARPi-FL accumulation using a fluorescence endoscope. Whole excised FaDu tumors were imaged using a confocal laser endomicroscope featuring a flexible confocal microprobe with a resolution up to 1.4 µm. FaDu tumor, mouse tongue, and muscle have been imaged 90 minutes post-injection of 150 nmol PARPi-FL (30% PEG300 in PBS) following sacrifice of the animals. Images are representative frames within real-time video recording of the organs at 488 nm laser excitation. Same window/leveling has been applied in all images. The fluorescence signal has been converted to an intensity scale.

Supplementary Table S1:

Characteristic	No. of patients	Percent (%)
Age at diagnosis		
Median (range)	63.2 (34.6 - 78.8)	-
Sex		
Male	6	50
Female	6	50
Tumor stage		
Premalignant	3	25
T2	3	25
T3	3	25
T4	3	25
Nodal Status		
N0	4	33
N1	2	17
N2a	0	0
N2b	6	50
Differentiation		
Well	2	17
Moderate to well	2	17
Moderate	5	42
Poorly	1	8
Unknown	1	8
Surgery (first-line therapy)		
No	0	0
Yes	12	100
